# Supplementary material for: No evidence of effects or interaction between the widely used herbicide, glyphosate, and a common parasite in bumble bees
Source: PeerJ. 2021 Nov 17;9:e12486. doi: 10.7717/peerj.12486 (PMC8605762; doi:10.7717/peerj.12486)
Supplement: Supplemental Information 1 [file peerj-09-12486-s001.docx]

Supplementary Information: No evidence of effects or interaction between widespread herbicide, glyphosate, and Trypanosome parasite, *Crithidia bombi,* in Bumble Bees, *Bombus terrestris*.

*Edward A. Straw^1*^, Mark J. F. Brown^1^*

*Corresponding Author: EdwardAStraw@gmail.com

^1^Centre for Ecology, Evolution & Behaviour, Department of Biological Sciences, School of Life Sciences and the Environment, Royal Holloway University of London, Egham, Surrey, TW20 0EX, UK.

| ***C. bombi* only**  10,000 *C. bombi* cells per worker  *n* = 23 |
| --- |
| **Glyphosate and *C. bombi***  10,000 *C. bombi* cells per worker  200μg per worker  *n* = 21 |

Supplemental Table 1. Experiment 1: Modified OECD version 1 treatment conditions and number of bees per treatment.

Supplemental Table 2. Experiment 2: Modified OECD version 2 treatment conditions and number of bees per treatment.

| **Control**  *n* = 45 | ***C. bombi* only**  10,000 *C. bombi* cells per worker  *n* = 32 | **Positive control**  4µg dimethoate per worker  *n* = 36 |
| --- | --- | --- |
| **Glyphosate only**  200μg per worker  *n* = 40 | **Glyphosate and *C. bombi***  10,000 *C. bombi* cells per worker  200μg per worker  *n* = 34 |  |

Supplemental Table 3. Experiment 3: Modified OECD version 3: Long Term Survival treatment conditions and number of bees per treatment.

| **Control**  *n* = 51 | ***C. bombi* only**  10,000 *C. bombi* cells per worker  *n* = 50 | **Positive control**  4µg dimethoate per worker  *n* = 30 |
| --- | --- | --- |
| **Glyphosate only**  200μg per worker  *n* = 44 | **Glyphosate and *C. bombi***  10,000 *C. bombi* cells per worker  200μg per worker  *n* = 47 |  |

Supplemental Table 4. Experiment 4: Microcolony Exposure- Acute treatment conditions and number of bees per treatment. *n*_s_ - *n*_e_ does not always equal the number of deaths, because a small number of bees escaped the cages, or did not feed on the solutions.

| **Control**  Microcolonies *n_m_* = 8  Number of bees alive at start of experiment *n_s_* = 64  Number of bees alive at end of experiment *n_e_* = 62 | ***C. bombi* only**  10,000 *C. bombi* cells per worker  Microcolonies *n_m_* = 11  Number of bees alive at start of experiment *n_s_* = 88  Number of bees alive at end of experiment *n_e_* = 74 |
| --- | --- |
| **Glyphosate only**  200μg per worker  Microcolonies *n_m_* =9  Number of bees alive at start of experiment *n_s_* = 72  Number of bees alive at end of experiment *n_e_* = 62 | **Glyphosate and *C. bombi***  10,000 cells per worker  200μg per worker  Microcolonies *n_m_* =10  Number of bees alive at start of experiment *n_s_* = 80  Number of bees alive at end of experiment *n_e_* = 64 |

Supplemental Table 5. Experiment 5: Microcolony Exposure- Chronic treatment conditions and number of bees per treatment. *n*_s_ - *n*_e_ does not always equal the number of deaths, because a small number of bees escaped the cages, or did not feed on the solutions.

| **Control**  Microcolonies *n_m_* = 8  Number of bees alive at start of experiment *n_s_* = 64  Number of bees alive at end of experiment *n_e_* = 45 | ***C. bombi* only**  10,000 cells per worker  Microcolonies *n_m_* = 8  Number of bees alive at start of experiment *n_s_* = 64  Number of bees alive at end of experiment *n_e_* = 44 |
| --- | --- |
| **Glyphosate only**  200μg per worker  *n* = microcolonies 8  Number of bees alive at start of experiment *n_s_* = 64  Number of bees alive at end of experiment *n_e_* = 46 | **Glyphosate and *C. bombi***  10,000 cells per worker  200μg per worker  *n* = microcolonies 8  Number of bees alive at start of experiment *n_s_* = 64  Number of bees alive at end of experiment *n_e_* = 42 |

Supplementary Tables 6. Modified Ecotoxicological Protocol OECD 247: Long Term Survival, Mortality: The results of the model selection process for each analysis using the package ‘MuMIn’ (Bartoń 2020). Predictors, AIC, ∆AIC from the Best Model, AIC Weight and whether the model was included in the final parameter estimates are all presented.

| Model Name | Predictors | AIC | ∆AIC from best model | AIC Weight | Included in Final Model Set |
| --- | --- | --- | --- | --- | --- |
| FM | Colony of Origin | 100.4 | 6.18 | 0.039 | No |
| M1 | Treatment, Colony of Origin | 98.6 | 4.34 | 0.098 | Yes |
| M0 | Treatment, Bee Weight, Colony of Origin | 94.3 | 0.00 | 0.862 | Yes |

Supplementary Tables 7. Experiment four: Microcolony Exposure- Acute Exposure, Reproduction. The results of the model selection process for each analysis using the package ‘MuMIn’ (Bartoń 2020). Predictors, AIC, ∆AIC from the Best Model, AIC Weight and whether the model was included in the final parameter estimates are all presented.

| Model Name | Predictors | AIC | ∆AIC from best model | AIC Weight | Included in Final Model Set |
| --- | --- | --- | --- | --- | --- |
| FM | Treatment, Bee Weight, Bees Alive at End, Colony of Origin | 7.2 | 27.35 | 0.000 | Yes |
| M1 | Treatment, Bee Weight, Colony of Origin | -1.8 | 18.32 | 0.000 | No |
| M2 | Treatment, Bees Alive at End, Colony of Origin | 10.3 | 30.39 | 0.000 | No |
| M3 | Treatment, Colony of Origin | 2.7 | 22.79 | 0.000 | No |
| M4 | Bee Weight, Colony of Origin | -20.1 | 0 | 0.991 | Yes |
| M5 | Bees Alive at End, Colony of Origin | -5.1 | 14.97 | 0.001 | No |
| M0 | Colony of Origin | -10.5 | 0.63 | 0.008 | No |

Supplementary Tables 8. Experiment four: Microcolony Exposure- Acute Exposure, Parasite Intensity. The results of the model selection process for each analysis using the package ‘MuMIn’ (Bartoń 2020). Predictors, AIC, ∆AIC from the Best Model, AIC Weight and whether the model was included in the final parameter estimates are all presented.

| Model Name | Predictors | AIC | ∆AIC from best model | AIC Weight | Included in Final Model Set |
| --- | --- | --- | --- | --- | --- |
| FM | Treatment, Microcolony, Colony of Origin | 2742.6 | 2.13 | 0.177 | Yes |
| M1 | Treatment, Colony of Origin | 2740.4 | 0 | 0.513 | Yes |
| M2 | Treatment, Microcolony | 2741.4 | 1.01 | 0.309 | Yes |
| M0a | Colony of Origin | 2754.4 | 14.01 | 0.000 | No |
| M0b | Microcolony | 2755.7 | 15.22 | 0.000 | No |

Supplementary Tables 9. Experiment four: Microcolony Exposure- Chronic Exposure, Reproduction. The results of the model selection process for each analysis using the package ‘MuMIn’ (Bartoń 2020). Predictors, AIC, ∆AIC from the Best Model, AIC Weight and whether the model was included in the final parameter estimates are all presented.

| Model Name | Predictors | AIC | ∆AIC from best model | AIC Weight | Included in Final Model Set |
| --- | --- | --- | --- | --- | --- |
| FM | Treatment, Bee Weight, Bees Alive at End, Colony of Origin | 5.7 | 24.57 | 0.000 | Yes |
| M1 | Treatment, Bee Weight, Colony of Origin | -0.9 | 17.93 | 0.000 | No |
| M2 | Treatment, Bees Alive at End, Colony of Origin | 3.6 | 22.48 | 0.000 | No |
| M3 | Treatment, Colony of Origin | -4.2 | 14.64 | 0.000 | No |
| M4 | Bee Weight, Colony of Origin | -17.0 | 1.84 | 0.279 | Yes |
| M5 | Bees Alive at End, Colony of Origin | -11.8 | 7.03 | 0.021 | No |
| M0 | Colony of Origin | -18.9 | 0.00 | 0.699 | Yes |

Supplementary Tables 10. Experiment four: Microcolony Exposure- Chronic Exposure, Sucrose Consumption. The results of the model selection process for each analysis using the package ‘MuMIn’ (Bartoń 2020). Predictors, AIC, ∆AIC from the Best Model, AIC Weight and whether the model was included in the final parameter estimates are all presented.

| Model Name | Predictors | AIC | ∆AIC from best model | AIC Weight | Included in Final Model Set |
| --- | --- | --- | --- | --- | --- |
| FM | Treatment, Bee Weight, Microcolony, Colony of Origin | -224.0 | 63.77 | 0.000 | No |
| M1 | Treatment, Microcolony, Colony of Origin | -229.3 | 72.01 | 0.000 | No |
| M2 | Treatment, Colony of Origin | -215.7 | 22.48 | 0.000 | No |
| M3 | Treatment, Microcolony | -229.0 | 58.73 | 0.000 | No |
| M0a | Bee Weight, Microcolony, Colony of Origin | -282.1 | 5.59 | 0.053 | Yes |
| M0b | Bee Weight, Colony of Origin | -267.6 | 20.12 | 0.000 | No |
| M0c | Bee Weight, Microcolony | -283.0 | 4.73 | 0.081 | Yes |
| M0d | Microcolony, Colony of Origin | -287.7 | 0.00 | 0.866 | Yes |

Supplementary Tables 11. Experiment four: Microcolony Exposure- Chronic Exposure, Parasite Intensity. The results of the model selection process for each analysis using the package ‘MuMIn’ (Bartoń 2020). Predictors, AIC, ∆AIC from the Best Model, AIC Weight and whether the model was included in the final parameter estimates are all presented.

| Model Name | Predictors | AIC | ∆AIC from best model | AIC Weight | Included in Final Model Set |
| --- | --- | --- | --- | --- | --- |
| FM | Treatment, Microcolony, Colony of Origin | 1683.6 | 1.69 | 0.239 | Yes |
| M1 | Treatment, Colony of Origin | 1684.0 | 2.04 | 0.202 | Yes |
| M2 | Treatment, Microcolony | 1681.9 | 0.00 | 0.558 | Yes |
| M0a | Colony of Origin | 1697.6 | 15.68 | 0.000 | No |
| M0b | Microcolony | 1699.3 | 17.36 | 0.000 | No |
| M0c | Microcolony, Colony of Origin | 1700.0 | 18.07 | 0.000 | No |
